# CD19: a promising target for systemic sclerosis

**DOI:** 10.3389/fimmu.2024.1454913

**Published:** 2024-10-03

**Authors:** Kazuhiro Komura

**Affiliations:** Department of Dermatology, Kanazawa Red Cross Hospital, Japanese Red Cross Society, Kanazawa, Ishikawa, Japan

**Keywords:** systemic sclerosis, fibrosis, CD19, CD20, therapy

## Abstract

Systemic sclerosis (SSc) is an autoimmune disease characterized by immune dysregulation, vascular damage, and fibrosis. B cells play a significant role in SSc through autoantibody production, cytokine secretion, and T cell regulation. Autoantibodies like anti-topoisomerase I and anti-RNA polymerase III are specific to SSc and linked to clinical features such as skin and lung involvement. B cell depletion therapies, particularly anti-CD20 antibodies like rituximab, have shown benefits in treating SSc, improving skin and lung disease symptoms. However, CD19, another B cell marker, is more widely expressed and has emerged as a promising target in autoimmune diseases. CD19-targeted therapies, such as CAR T cells and Uplizna^®^ (inebilizumab), have demonstrated potential in treating refractory autoimmune diseases, including SSc. Uplizna^®^ offers advantages over rituximab by targeting a broader range of B cells and showing higher efficacy in specific patient subsets. Clinical trials currently investigate Uplizna^®^’s effectiveness in SSc, particularly in severe cases. While these therapies offer hope, long-term safety and efficacy remain unknown. SSc is still a complex disease, but advancing B cell-targeted treatments could significantly improve patient outcomes and knowledge about the pathogenesis.

## Introduction

Systemic sclerosis (SSc) or scleroderma is characterized by immune dysregulation, obliterative microvasculopathy, and fibrosis, significant causes of mortality throughout the body (1, 2).

B cells are implicated in the pathogenesis of SSc through several mechanisms, including autoantibody production, cytokine secretion, antigen presentation, and regulation of T cell responses (3, 4). Autoantibodies are a hallmark of SSc and can be detected in more than 90% of patients (5–9). They are associated with distinct clinical features, such as skin involvement, lung fibrosis, or pulmonary arterial hypertension (8). Some autoantibodies, such as anti-topoisomerase I, anti-RNA polymerase III, and anti-centromere, are highly specific for SSc and can be used as diagnostic markers (8). B cells also secrete pro-inflammatory and pro-fibrotic cytokines, such as interleukin-6 (IL-6), tumor necrosis factor-alpha (TNF-α), and transforming growth factor-beta (TGF-β), which contribute to the activation of fibroblasts and endothelial cells (4, 10). Moreover, B cells can act as antigen-presenting cells and stimulate T cell responses, further enhancing immune dysregulation and tissue damage in SSc (10). Finally, B cells can modulate the immune system through regulatory B cells (Bregs), which produce anti-inflammatory cytokines, such as IL-10 and IL-35, and suppress effector T cells (11). However, the function and frequency of Bregs are impaired in SSc, leading to a loss of immune tolerance and homeostasis (12). In several studies, B cell depletion therapy with anti-CD20 antibodies has shown some efficacy in improving skin and lung manifestations of SSc. Other B cell targets, such as CD19, are also being explored as potential therapeutic options for SSc.

## Anti-CD20 antibody therapy for SSc

CD20 is a transmembrane protein expressed on most B cells, except for stem cells and plasma cells. It is involved in the activation and differentiation of B cells and can modulate B cell receptor signaling and calcium flux (13). Anti-CD20 antibodies can bind to CD20 and induce B cell depletion through ADCC, CDC, or direct apoptosis (14–16). In experimental models of SSc, anti-CD20 antibodies have shown anti-fibrotic and anti-inflammatory effects by reducing collagen deposition, fibroblast activation, and cytokine production (17). They have also decreased the levels of autoantibodies in SSc mice (17). In clinical trials, anti-CD20 antibodies have demonstrated some benefits for patients with SSc, especially for skin and lung involvement. Rituximab, a chimeric monoclonal antibody against CD20, was efficacious for both skin sclerosis and ILD in SSc in case reports (18, 19), open-label trials (20) and small-scale RCTs (21, 22). In a case-control study of 25 patients with severe SSc (MRSS >16), rituximab treatment significantly improved skin sclerosis (mean ± SEM MRSS, from 26.6 ± 1.4 to 20.3 ± 1.8; P = 0.0001) after six months (range, 5–9) (23). Additionally, nine SSc patients with ILD and rituximab preserved %FVC (mean ± SEM %FVC, from 60.6 ± 2.4 to 61.3 ± 4.1; P = 0.5) (20). Consistently, rituximab significantly reduced MRSS while retaining pulmonary function at 12 months in an open-label, uncontrolled study of 20 patients (24). A randomized controlled trial of rituximab for SSc was conducted with 56 patients (21). They received either rituximab 375 mg/m2 or a placebo by infusion once a week for four weeks. The primary outcome was the difference in skin thickening measured by MRSS at 24 weeks after the treatment. Rituximab significantly reduced 6.30 MRSS points (2.14 increase in placebo; difference −8.44 [95% CI −11.00 to 5.88]; p < 0.00001). Thus, rituximab was the first drug for SSc to show efficacy in a double-blind, randomized, and placebo-controlled trial with skin sclerosis at the primary endpoint. Based on the results, rituximab was approved for SSc in Japan in 2021.

One of the main concerns about rituximab therapy for SSc is long-term safety, especially the risk of infections and malignancies. Although anti-CD20 antibodies have been used for over a decade in other autoimmune diseases, such as rheumatoid arthritis and systemic lupus erythematosus, the data on their long-term safety in SSc are still limited (25, 26). A recent systematic review and meta-analysis of 14 studies with 597 SSc patients who received rituximab showed that the overall mean rate for rituximab-related severe adverse events rate was 12.1% (p=0.733, I^2^ = 0%) throughout treatment, which was acceptable and controllable for oncologist and rheumatologist to manage (27).

Further limitations and challenges in using anti-CD20 antibodies for SSc are mode of actions during depleting B cells. An emerging strategy we can consider is the use of obinutuzumab in the treatment of SSc as well as SLE (28). Obinutuzumab is a humanized type II monoclonal anti-CD20 antibody, known for its more effective B cell depletion than rituximab (29, 30). In the phase 2 randomized and placebo-controlled trial, obinutuzumab demonstrated efficacy in achieving both complete and overall renal responses at week 52 when combined with mycophenolate and corticosteroids in patients with lupus nephritis (31). The difference between newly generated type II antibody obinutuzumab and rituximab depends on antibody internalization, Fc-Fc Receptor interactions, CDC, and homotypic adhesions/intracellular signaling within experimental studies in SLE and RA (16). Obinutuzumab demonstrates efficacy in low complement and rituximab-resistant SLE, since obinutuzumab depletes B cells in a complement-independent manner (28). Thus, more studies are needed to identify the best candidates, the most effective regimen, and the potential predictors of anti-CD20 therapy within SSc pathogenesis.

## CD19 and CD20

CD19 and CD20 are molecules expressed on B cells’ surface but have different functions and roles in immune regulation (13). CD20 is a transmembrane protein that forms homodimers and acts as a calcium channel for B cell activation. CD20 is involved in the development and maturation of B cells, and its expression is relatively constant throughout the B cell lineage, starting from late pre-B lymphocytes (pro-B lymphocytes do not express it) and its expression is lost in terminally differentiated plasmablasts and plasma cells (32). While CD20 is generally not expressed in mature plasmablasts, it is present in early plasmablasts under certain circumstances, although it remains uncertain in patients with SSc (33). CD20 is also expressed on a small subpopulation of T cells (34). CD20 is the target of several monoclonal antibodies, such as rituximab, that deplete B cells and modulate their functions in autoimmune diseases.

CD19, on the other hand, is a glycoprotein that forms a multimolecular cell surface signal-transduction complex, including CD21 and CD81 (35). In contrast to a negative regulator of CD22, CD19 acts as a positive response regulator for BCR signaling (36, 37). CD19 regulates the threshold and intensity of BCR signaling and thus influences B cells’ differentiation, antibody production, and memory formation (38). CD19 expression is variable and depends on the activation state and subtype of B cells, and it is downregulated upon plasma cell differentiation (**Table 1**) (13, 38–40). CD19 is expressed at high levels by all mature B cells, memory B cells, and plasmablasts, while some pre-B cells and plasma cells express CD19. Plasmablasts are transient antibody-secreting cells those are short-lived with high proliferation rate. Plasma cells, having a distinct morphology characterized by an eccentrically placed nucleus with coarse chromatin arranged in a clock face pattern and abundant cytoplasm immunoglobulin inclusion, can be long-lived secreting antibodies in the bone marrow and at the inflammatory sites (41–43). Thus, CD19 is widely expressed on B cell linage, including CD20-negative pre-B cells, CD20-negative plasmablasts, and CD20-negative plasma cells, in addition to CD20-positive B cells.

**Table 1:** Cell surface CD19 and CD20 expression during B-cell development.

Expression	Stem cell	Pro-B cell	Pre-B cell	Immature B cell	Mature B cell	Memory B cell	Plasmablasts	Plasma cell
CD20								
CD19								

Red and pink indicate positivity and partial positivity of CD20, respectively. Partial and high CD19 expression are indicated in light green and dark green, respectively.

When comparing the effects of two monoclonal antibodies, anti-CD19 and anti-CD20, on an animal model of multiple sclerosis, anti-CD19 is more effective than anti-CD20 in reducing autoantibodies and plasma cells that cause inflammation and damage in the nervous system. The result of the study identifies a subset of plasma cells that express CD19 but not CD20, which are resistant to anti-CD20 therapy and may be responsible for residual disease activity (41).

## Potential role of CD19 in SSc pathogenesis

CD19 expression and function are augmented in various autoimmune diseases, including SSc (44, 45). On the other hand, genetic deficiency in CD19 reveals primary immunodeficiency syndrome (46). CD19 expression is increased in peripheral B cells from patients with SSc compared to healthy controls and correlates with disease activity, skin fibrosis, and autoantibody levels (44). Genetic variants of CD19 have been associated with augmented CD19 expression levels, increased susceptibility, and different clinical phenotypes in SSc (47, 48). In the experimental SSc model, CD19 ligation induced augmented tyrosine phosphorylation of Vav and intracellular Ca responses, major effector responses downstream of CD19 signaling in B cells (10, 49). Experimental SSc models also demonstrated CD19-associated skin sclerosis, pulmonary fibrosis, and autoimmunity, since these phenotypes were normalized in CD19 deficiency (10, 50–53). In addition to autoantibody production, up-regulated CD19 on B cells may also play a role in local inflammation since homing receptor CXCR3 was positive on autoreactive B cells at the inflammatory sites in SLE, RA joint, and experimental SSc-pulmonary fibrosis (52, 54, 55). Thus, enhanced CD19 signaling induces hyperreactivity of B cell responses at the site of inflammation and enhances phenotypes of autoimmune diseases, while CD19 deficiency causes immunodeficiency and underscoring SSc phenotype.

## Chimeric antigen receptor T therapy in autoimmune diseases

CAR T therapy is a novel and promising approach to target malignant B cells that escape conventional B cell depletion (56). CAR T cells are genetically engineered T cells that express a synthetic receptor that recognizes a specific antigen on the surface of B cells, such as CD19, CD20, or CD22, generated to treat B cell malignancy (57, 58). Upon binding to the antigen, CAR T cells activate and kill the malignant and activated B cells while sparing other immune cells that do not express the antigen. CAR T therapy has successfully treated B cell malignancies, such as acute lymphoblastic leukemia, chronic lymphocytic leukemia, and non-Hodgkin’s lymphoma (56). Recently, CAR T therapy has also been explored in treating autoimmune diseases, such as SLE, dermatomyositis, multiple sclerosis, and SSc (59–62) (**Table 2**). In these cases, CAR T cells were directed against CD19. The results showed that CAR T therapy induced a rapid and sustained depletion of circulating B cells and a complete clinical and serological remission of autoimmune diseases without causing severe adverse events or infections (63, 66). In a case series, four patients with refractory SSc were significantly improved by single CD19 CAR T cell infusion after preconditioning with fludarabine cyclophosphamide (63). All SSc patients had a decrease in MRSS (-9 points, ranging from -17 to -7) and the median change in the EUSTAR activity index (-4.2 points, ranging from -4.7 to -2.3) at least six months of follow-up. These responses looked greater than CD20-directed rituximab, as previously described. SSc-associated scl-70 autoantibody levels were also decreased in all patients with SSc after CD19 CAR T therapy. As a result, all patients successfully discontinued immunosuppressive medications during the follow-up after CD19 CAR. In other case series, CD19-targeted CAR-T therapy achieved 50% improvement in MRSS after six months in two patients with SSc (62). However, CAR T therapy’s long-term effects and safety in autoimmune diseases remain unknown. In an experimental animal model (fos-related antigen-2 transgenic mouse), CD19 CAR therapy paradoxically worsens pulmonary fibrosis (65). Therefore, careful discussion is needed about the indication of CAR-T therapy for SSc. Moreover, CAR T requires specific facilities for genomics and over 200,000$ cost. There are still challenges in making these therapies widely accessible for patients with autoimmune diseases.

**Table 2:** Human and animal studies of CD19 targeting therapies in SSc.

Study Type	Authors/Year	Phase	Model	Therapy	Outcome
**Human Study**	Wang X et al., 2024 (62)	Case reports	Human (Systemic Sclerosis Patients)	Anti-CD19 CAR-T Cells	Reduction in skin score (-20 MRSS after 6 months) and lung fibrosis in 2 SSc patients.
**Human Study**	Muller F 2024 (63)	Case reports	Human (Systemic Sclerosis Patients)	Anti-CD19 CAR-T Cells	Decrease in B-cell activation and decreased skin score (-9 MRSS after 6 months) in 4 SSc patients
**Human Study**	Schiopu E et al., 2016 (64)	Phase I	Human (Systemic Sclerosis Patients)	Anti-CD19 MonoclonalAb (Inebilizumab-cdon)	A low-dose anti-CD19 ab decreased B-cell activation and skin score (-5.4 MRSS at day 85) in 24 SSc patients.
**Animal Study**	Avouac J et al., 2024 (65)	Animal study	Mouse (Fra-2 Tg mice)	Anti-CD19 CAR-T Cells +Anti-CD20 Monoclonal Ab	Worsening lung fibrosis

## Uplizna ^®^ (inebilizumab) therapy in autoimmune diseases and SSc

Uplizna^®^ is an afucosylated IgG1 monoclonal antibody that binds to CD19 and depletes B cells (67), which are involved in the immune response in autoimmune diseases, including neuromyelitis optica spectrum disorder (NMOSD); a rare and severe neurological disease. A clinical trial showed that inebilizumab reduced the risk of NMOSD attacks by more than 70% compared to placebo without causing severe side effects. Uplizna^®^ could be an effective treatment for NMOSD patients (68). Uplizna^®^ was approved by the FDA in 2020 as the first drug for the treatment of NMOSD patients with anti-aquaporin 4 Ab. Of note, in a *post hoc* analysis, Uplizna^®^ decreased ~10-fold attack rate in 13 NMOSD patients with prior rituximab use (69).

Uplizna^®^ appeals advantages in comparison to rituximab (67). First, Uplizna^®^ targets CD19 in contrast to CD20-targeted rituximab (**Table 1**). CD19 is expressed on pro-B cells, autoreactive plasmablasts, plasma cells, and CD20+B cells. Targeting immature cells, Uplizna^®^ enables B cell depletion with fewer injections and avoids B cell recovery. Autoreactive plasmablasts and plasma cell depletion reduce autoantibody production and other pathological B cell activities. Secondly, removing fucose from the Fc region results in approximately tenfold increased affinity for the activating Fc gamma receptor IIIA. It significantly enhances natural killer cell-mediated depletion of B cells via ADCC and antibody-dependent cellular phagocytosis mechanisms (70, 71). The relevance of ADCC and antibody-mediated phagocytosis is now well established in contrast to CDC and apoptosis (72). Thus, Uplizna^®^ demonstrates maximum ADCC activities, while rituximab showed modest CDC and limited ADCC activities. This results in Uplizna^®^ efficiently depleting B cells even in approximately 40% of the homozygous population for the genetic risk factor of antibody therapy resistance (73, 74). Thirdly, Uplizna^®^ has a humanized structure. Humanized structures are associated with reduced immunogenicity, improved tolerability, and decreased potential for infusion-related reactions compared with chimeric structure rituximab. Accordingly, the anti-drug antibodies positive rate was low (9.8%) in Uplizna-treated MNOSD (75). Thus, Uplizna^®^ is an efficient and safe CD19-target drug.

On the other hand, Uplizna^®^ may activate the Hepatitis B virus and latent tuberculosis, similar to other immunosuppressive therapies. Therefore, target patients require screening for these infections before starting the drug infusion. In addition, with continued Uplizna^®^ effect, there may be progressive and prolonged hypogammaglobulinemia or a decline in total and individual immunoglobulin levels. In NMOSD patients treated with Uplizna^®^ for ≥ 4 years, 46.7% of patients had normal IgG levels (≥ 700 mg/dL), 29.3% had mild hypogammaglobulinaemia (500 to < 700 mg/dL), 20% had moderate (300 to < 500 mg/dL) and 4% had severe (< 300 mg/dL). Low IgG titres were not associated with severe infection, although the number of patients was small (76). The therapy requires monitoring the level of immunoglobulins at the beginning, during, and after treatment discontinuation with Uplizna^®^ until B-cell repletion, especially in patients with infections and initial lower IgM levels. Because hypogammaglobulinemia after rituximab treatment was largely restricted to the IgM class and associated with low baseline levels (77).

MITIGATE (78), a placebo-controlled phase 3 trial testing Uplizna^®^ efficacy and safety on IgG4-related disease (IgG4-RD; NCT04540497), met its primary endpoint, showing a statistically significant 87% reduction in the risk of IgG4-RD flare compared to placebo (Hazard Ratio 0.13, p<0.0001) during the 52-week placebo-controlled period. All critical secondary endpoints were also met. The overall safety results during the placebo-controlled trial period were consistent with Uplizna^®^’s known safety profile.

Uplizna^®^ has also been investigated in other autoimmune diseases like myasthenia gravis (NCT04524273). However, its role in SSc has not yet been fully explored. Given the similarities between NMOSD and SSc regarding B cell involvement, vascular damage, and autoimmunity, Uplizna^®^ may have therapeutic potential for SSc patients, precisely in the CD19-overexpressing SSc subset (64, 79, 80). Furthermore, Uplizna^®^ has advantages in B cell-directed therapy over rituximab, which has been approved for SSc treatment in Japan (81). Therefore, clinical trials are initiated to evaluate the safety and efficacy of Uplizna^®^ in SSc, especially in those with severe skin and lung involvement.

## Conclusion

SSc is a heterogeneous and complex disease that affects multiple organs and systems. However, CD19 on B cells plays a vital role in the pathogenesis of SSc, and B cell-targeted therapies have shown promising results in some patients. Uplizna^®^ is a novel B cell-depleting agent approved for NMOSD with some similarities to SSc. The potential of Uplizna^®^ for treating SSc has been initiated in clinical trials.
